# Sarcopenia and Physical Function in Obstructive Sleep Apnea: A Single‐Centre Cross‐Sectional Study (The SOSA Study)

**DOI:** 10.1111/crj.70201

**Published:** 2026-06-05

**Authors:** Ahmad J. Abdulsalam, Renu Ambardar, Mohammad Abdulsalam, Sulaiman Khadadah, Sarah Alkandari, Abdulrahman Abdulsalam, Diaa Shehab, Murat Kara, Levent Özçakar

**Affiliations:** ^1^ Department of Physical Medicine and Rehabilitation Mubarak Al Kabeer Hospital Jabriya Kuwait; ^2^ Sleep Lab, Respiratory Unit, Department of Internal Medicine Mubarak Al Kabeer Hospital Jabriya Kuwait; ^3^ Royal College of Surgeons in Ireland University Busaiteen Bahrain; ^4^ Faculty of Medicine Kuwait University Kuwait City Kuwait; ^5^ Department of Physical and Rehabilitation Medicine Hacettepe University Medical School Ankara Türkiye

**Keywords:** algorithms, body composition, muscle, obstructive, quadriceps muscle, sarcopenia, sleep apnea, skeletal, ultrasonography

## Abstract

**Introduction:**

Whether obstructive sleep apnea (OSA) severity is independently associated with sarcopenia, beyond the effects of age, obesity and sex, has not been established in a single‐centre cohort using standardised ultrasound‐based assessment. We examined sarcopenia prevalence and its components across OSA severity strata in a Kuwaiti cohort using the ISarcoPRM sarcopenia algorithm.

**Methods:**

Cross‐sectional within‐cohort analysis of 110 adults aged 50 years or older with confirmed OSA (apnea‐hypopnea index [AHI] 5 or more events/h by Level 3 portable monitoring; SomnoTouch, Somnomedics, Germany), stratified as mild (AHI 5–14.99, *n* = 28), moderate (AHI 15–29.99, *n* = 39) or severe (AHI 30 or more, *n* = 43). Sarcopenia was assessed using the ISarcoPRM algorithm: quadriceps muscle thickness by ultrasound, Sonographic Thigh Adjustment Ratio (STAR), handgrip strength (Jamar dynamometer) and chair stand test (CST).

**Results:**

Demographic and comorbidity profiles were balanced across severity groups (all *p* > 0.05). Quadriceps muscle thickness, STAR and handgrip strength did not differ significantly across severity strata (all Kruskal–Wallis *p* > 0.05). CST time showed a significant gradient across severity strata (Kruskal–Wallis *p* = 0.047), and both AHI and ODI correlated modestly with CST time (*r* = +0.209, *p* = 0.029 and *r* = +0.203, *p* = 0.034, respectively). Sarcopenia prevalence was 21.4%, 30.8% and 34.9% in mild, moderate and severe OSA, respectively, with no significant trend (Cochran–Armitage *p* = 0.237). Age (OR 1.12 per year, 95%CI 1.05–1.19, *p* < 0.001) and BMI (OR 1.10 per kg/m2, 95%CI 1.02–1.18, *p* = 0.009) were the independent predictors of sarcopenia; OSA severity was not (adjusted OR 1.19, 95%CI 0.65–2.18, *p* = 0.577). Low STAR prevalence was 83.6%, driven by the high‐obesity burden in this cohort and the origin of STAR cut‐offs in a lower BMI Turkish reference population.

**Conclusion:**

In this Kuwaiti OSA cohort, age and BMI are the dominant determinants of sarcopenia, with no independent contribution from OSA severity. A modest association between OSA severity indices and CST time suggests that physical function may be more sensitive to OSA‐related changes than muscle mass per se. The near‐universal low STAR prevalence points to the need for population‐specific normative data in high‐obesity cohorts.

## Graphical abstract

In 110 Kuwaiti adults with confirmed OSA, severity was not independently associated with sarcopenia (adjusted OR 1.19, *p* = 0.577). Age and BMI were the dominant predictors. Chair stand time showed a significant gradient across severity strata (*p* = 0.047). Low STAR prevalence was 83.6%, reflecting a floor effect from Turkish‐derived cut‐offs in a high‐obesity Arabian Gulf cohort.

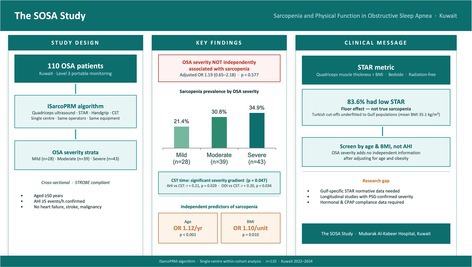

## 1 Introduction

Sarcopenia, defined as the progressive loss of skeletal muscle mass and function, is associated with impaired physical performance, increased fall risk, reduced quality of life and elevated mortality [1, 2]. Its prevalence rises from 5% to 13% in those aged 60–70 years to nearly 50% in individuals over 80 [3]. The European Working Group on Sarcopenia in Older People (EWGSOP2) incorporates appendicular skeletal muscle mass by dual‐energy X‐ray absorptiometry (DXA), handgrip strength and physical performance [1]. However, DXA has practical limitations, including cost, restricted access outside research centres and inability to characterise regional muscle quality or distribution. To address these limitations, the ISarcoPRM, special interest group of the International Society of Physical and Rehabilitation Medicine (ISPRM), has developed a point‐of‐care algorithm incorporating ultrasound‐measured quadriceps muscle thickness and the Sonographic Thigh Adjustment Ratio (STAR), calculated as quadriceps muscle thickness divided by BMI, alongside handgrip strength and the chair stand test (CST) [4]. Quadriceps muscle atrophy precedes declines in whole‐body appendicular muscle mass, making this region a sensitive early sarcopenia target [4, 5]. Ultrasound is non‐invasive, portable, radiation‐free and applicable at the bedside [5, 6].

Obstructive sleep apnea (OSA) affects an estimated 936 million adults worldwide [7] and is characterised by recurrent upper airway collapse, producing intermittent hypoxia, oxidative stress, systemic inflammation and metabolic dysregulation [8, 9]. These mechanisms may promote skeletal muscle loss through hypoxia‐induced reactive oxygen species drive muscle protein degradation [10], sleep fragmentation disrupts growth hormone secretion [11] and insulin resistance impairs muscle protein synthesis [12]. Population‐based data link sleep‐disordered breathing to adverse body composition [13, 14], and Piovezan et al. [15] demonstrated in the HypnoLaus cohort (*n* = 1902) that severe OSA (AHI > 30) was associated with low muscle strength (OR 2.36, 95%CI 1.29–4.31). Sun et al. [16] showed by Mendelian randomisation, however, that low grip strength may be causally upstream of OSA rather than only downstream, which complicates the assumed direction of effect. Prior observational studies are further limited by heterogeneous sarcopenia definitions, reliance on whole‐body imaging and insufficient adjustment for obesity.

The Sarcopenia in Obstructive Sleep Apnea (SOSA) study was conducted to evaluate sarcopenia and its components across OSA severity strata in a single Kuwaiti centre using the ISarcoPRM algorithm. The primary objective was to examine sarcopenia prevalence across mild, moderate and severe OSA. Secondary objectives were to evaluate associations between OSA severity indices (AHI and ODI) and muscle parameters and to identify independent predictors of sarcopenia and low STAR.

## 2 Methods

### 2.1 Setting and Study Design

This cross‐sectional study included adults aged 50 years or older with a confirmed diagnosis of OSA who were evaluated at the Sleep Unit of Mubarak Al Kabeer Hospital, Kuwait, between 2022 and 2024. All participants were recruited and assessed at a single centre by the same multidisciplinary team using the same equipment, eliminating intersite measurement variability. The study followed Strengthening the Reporting of Observational Studies in Epidemiology (STROBE) guidelines and was approved by the Kuwait Ministry of Health Ethics Committee (No. 2827/2025). Written informed consent was obtained from all participants.

### 2.2 Participants

**FIGURE 1:**
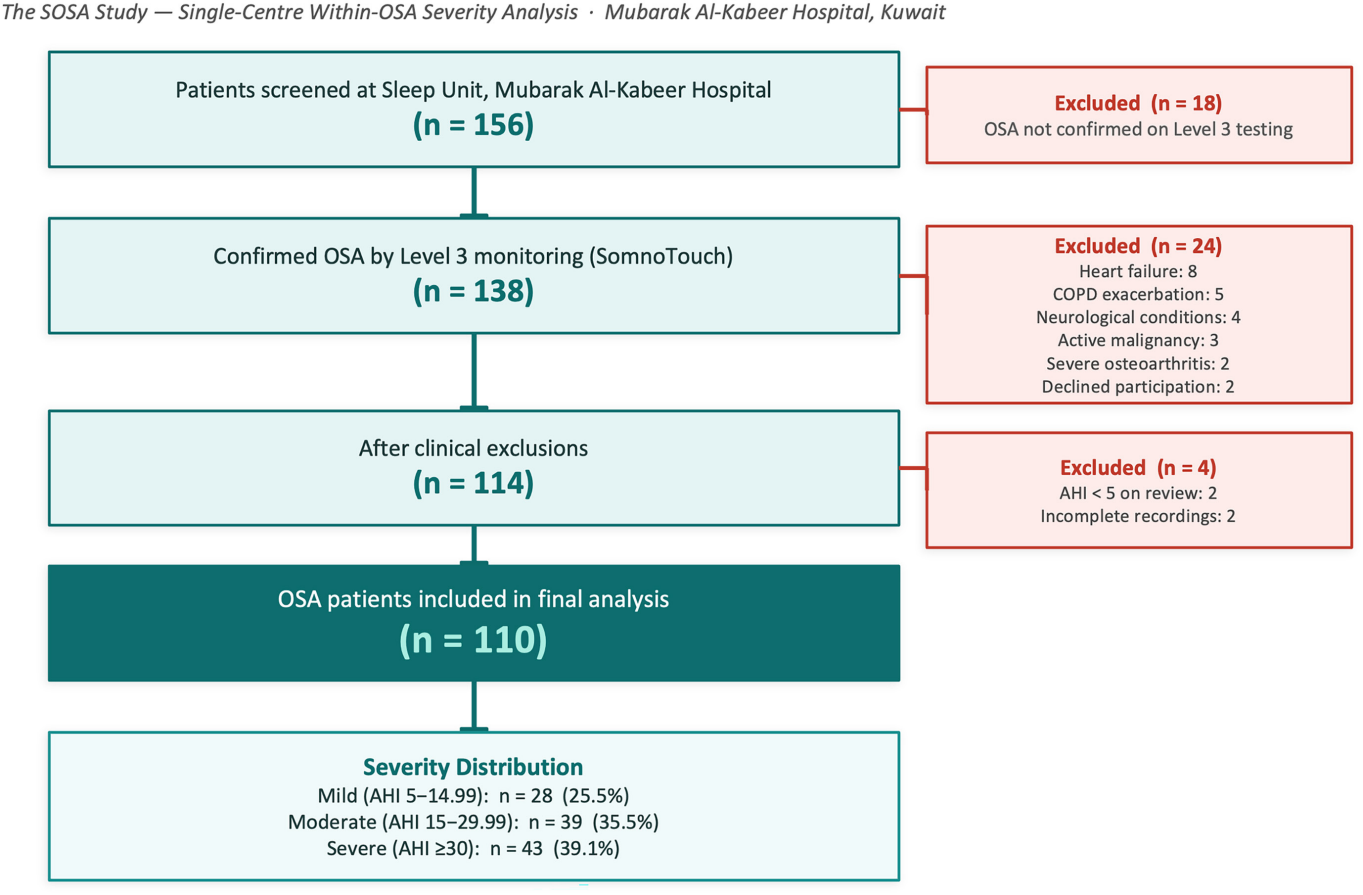
Study flow diagram. OSA and obstructive sleep apnea; AHI, apnea‐hypopnea index; COPD, chronic obstructive pulmonary disease.

Patients were eligible if they were aged 50 years or older and had a confirmed diagnosis of OSA (AHI 5 or more events/h). Exclusion criteria included heart failure, acute exacerbation of chronic obstructive pulmonary disease (COPD), prior stroke, chronic kidney or liver failure, Parkinson's disease, malignancy, neurological diseases affecting muscle function, major orthopaedic surgery within the preceding 12 months, severe osteoarthritis, active rheumatic disease and bedridden status. Patients with other respiratory diseases (including asthma, interstitial lung disease and nonexacerbating COPD) were not excluded unless the condition was associated with clinically significant functional impairment at the time of assessment, as judged by the examining physician. Of 156 patients screened, 138 had OSA confirmed by Level 3 monitoring. After exclusion of 24 patients (heart failure, *n* = 8; COPD exacerbation, *n* = 5; neurological conditions, *n* = 4; malignancy, *n* = 3; severe osteoarthritis, *n* = 2; declined participation, *n* = 2), two patients with AHI below 5 events/h on manual review, and two patients with incomplete portable monitoring recordings and 110 patients were included in the final analysis (Figure 1).

BMI was calculated as weight divided by height squared (kg/m2). Physical activity level was assessed using the International Physical Activity Questionnaire Short Form (IPAQ‐SF) and categorised as sedentary (no regular physical activity) or active. Smoking status, diabetes mellitus and hypertension were recorded from medical records. There were no missing values for any primary study variable.

### 2.3 Diagnosis of OSA

OSA was diagnosed using a Level 3 portable sleep monitoring system (SomnoTouch, Somnomedics, Germany). Recordings were manually scored by a certified sleep technician according to American Academy of Sleep Medicine (AASM) criteria [17]. Apnea was defined as complete cessation of airflow for 10 s or more; hypopnea as a 30%–90% reduction in flow accompanied by 3% or more oxygen desaturation. AHI was calculated as the number of events per hour of recording time. The oxygen desaturation index (ODI) was recorded as the number of 3% or more oxygen desaturation events per hour. OSA severity was classified as mild (AHI 5–14.99), moderate (AHI 15–29.99) or severe (AHI 30 or more).

### 2.4 Sarcopenia Assessment

Quadriceps muscle thickness was measured using an 8–10‐MHz linear probe (Logiq e, GE Medical Systems) at the midpoint between the anterior superior iliac spine and the superior border of the patella, with participants in the supine position. Adequate gel was applied to avoid tissue compression. All measurements were performed by two physicians with formal training in musculoskeletal ultrasonography, who served as the same operators throughout the study. Both operators followed standardised ultrasound‐based muscle assessment procedures per the 2020 SARCUS consensus [18]. Before study commencement, a feasibility pilot was performed in which both operators independently measured quadriceps thickness in a small convenience sample to standardise probe positioning, transducer pressure and image acquisition. A formal intrarater reliability coefficient (intraclass correlation coefficient or standard error of measurement) was not calculated within the study sample; this is noted as a limitation. Operators were not blinded to OSA severity category; severity classification was determined independently from sleep study reports before muscle assessment was performed.

STAR was calculated as quadriceps muscle thickness (mm) divided by BMI [4, 5]. Handgrip strength was measured using a Jamar dynamometer with subjects seated, shoulders adducted, and elbows at 90°; the highest of three attempts was recorded. The CST measured the time to complete five sit‐to‐stand repetitions without arm use [19]. The CST was selected as the functional performance measure within the ISarcoPRM algorithm because it directly evaluates lower extremity power relevant to the quadriceps muscles assessed by ultrasound, requires no equipment beyond a standard chair and has established normative values and test–retest reliability in older adults [19, 20].

Sarcopenia was diagnosed according to ISarcoPRM criteria [4]: low STAR (below sex‐specific cut‐offs from the Turkish reference cohort: below 1.0 for females, below 1.4 for males [21]) combined with either low handgrip strength (19 kg or less for females, 32 kg or less for males) or prolonged CST time (12 s or more). These cut‐offs were developed in a Turkish community cohort of 326 adults [21]; their applicability to Kuwaiti populations has not been validated, and this is acknowledged as a limitation (see Section 4).

### 2.5 Statistical Analysis

Analyses were performed in Python 3 (scipy 1.11, statsmodels 0.14). Continuous variables were compared across severity groups using the Kruskal–Wallis test; post hoc pairwise Mann–Whitney *U* tests were applied with Bonferroni correction for three comparisons (adjusted alpha 0.017). Categorical variables were compared using chi‐square tests. Trends in binary outcomes across ordinal severity strata were evaluated using the Cochran–Armitage trend test. Correlations between OSA severity indices (AHI and ODI) and muscle parameters were assessed by Spearman rank correlation. Binary logistic regression evaluated independent predictors of sarcopenia and low STAR, with OSA severity coded as an ordinal variable (0 = mild, 1 = moderate, 2 = severe), adjusted for age, sex, BMI, diabetes mellitus and hypertension; unadjusted and adjusted estimates are reported side by side. A sex‐by‐severity interaction term was added to test effect modification. As a sensitivity analysis, the logistic regression models were repeated with AHI entered as a continuous variable (per 10 events/h) in place of the ordinal severity term, to test whether the equal‐spacing assumption of the ordinal coding influenced the result. Standardised mean differences (SMDs) were calculated as balance diagnostics for Table 1 [22];|SMD| below 0.20, the conventional threshold for a small standardised difference, was considered to indicate adequate balance; any covariate exceeding this threshold was retained as an adjustment variable in the multivariable models. Sensitivity analyses excluded participants with severe obesity (BMI 40 kg/m2 or more). No multiple imputation was required as there were no missing values for any primary analysis variable. Statistical significance was set at two‐sided alpha 0.05 except where Bonferroni adjustment was applied.

**TABLE 1:** Participant characteristics by OSA severity (*n* = 110).

Variable	Overall (*n* = 110)	Mild (*n* = 28)	Moderate (*n* = 39)	Severe (*n* = 43)	*p*
Age, years	63.0 ± 9.2	61.1 ± 8.9	63.0 ± 8.6	64.1 ± 9.9	0.335
Male, sex	60 (54.5)	14 (50.0)	22 (56.4)	24 (55.8)	0.854
BMI, kg/m2	35.1 ± 7.1	35.0 ± 7.8	34.8 ± 6.6	35.3 ± 7.4	0.965
Obesity (BMI ≥ 30 kg/m^2^)	83 (75.5)	20 (71.4)	30 (76.9)	33 (76.7)	0.848
Morbid obesity (BMI ≥ 40 kg/m^2^)	23 (20.9)	5 (17.9)	9 (23.1)	9 (20.9)	0.874
Current smoker	41 (37.3)	7 (25.0)	14 (35.9)	20 (46.5)	0.182
DM	48 (43.6)	10 (35.7)	17 (43.6)	21 (48.8)	0.552
HT	66 (60.0)	15 (53.6)	22 (56.4)	29 (67.4)	0.431
Sedentary	77 (70.0)	21 (75.0)	28 (71.8)	28 (65.1)	0.644
AHI, events/h	29.9+/−19.6	10.2+/−2.8	22.0+/−4.5	49.9+/−15.7	< 0.001
ODI, events/h	27.0+/−21.4	7.7+/−3.5	19.5+/−6.5	46.4+/−21.4	< 0.001
|SMD|mild vs. severe*	—	Reference	—	Age 0.32, BMI 0.04, Sex 0.12, DM 0.27, HT 0.29	—

*Note:* Data are mean ± SD or *n* (%). Kruskal–Wallis or chi‐square as appropriate. Significant variables are shown as bold.

* Standardised mean differences [22];|SMD| < 0.20 indicates adequate balance. AHI, apnea‐hypopnea index; BMI, body mass index; DM, diabetes mellitus; HT, hypertension; ODI, oxygen desaturation index.

A sample size of 110 participants was available from the clinical database. Post hoc power calculations show that this sample provides approximately 60% power to detect the observed 13‐percentage‐point difference in sarcopenia prevalence between mild and severe OSA at two‐sided alpha 0.05; the study is therefore underpowered by conventional standards. The originally planned 15% prevalence difference (derived from published sarcopenia prevalence data in sleep‐disordered breathing populations [13, 14]), which informed the 104‐patient target, was closely approximated by the observed difference. Results should be treated as descriptive and hypothesis‐generating given the cross‐sectional design.

## 3 Results

### 3.1 Participant Characteristics

Of 110 participants included, 28 had mild, 39 moderate and 43 severe OSA. Mean age was 63.0 ± 9.2 years; 60 (54.5%) were male; mean BMI was 35.1 ± 7.1 kg/m2. All *p*‐values exceeded 0.05 for non‐AHI variables (Table 1). On the SMD metric, BMI and sex were well balanced (|SMD| 0.04 and 0.12), whereas age, diabetes and hypertension showed small imbalances above the 0.20 threshold (|SMD| 0.27 to 0.32); all five variables were included as covariates in the adjusted regression models, so the primary estimate for OSA severity is adjusted for these differences. AHI and ODI differed across groups by design (both *p* < 0.001).

### 3.2 Muscle Mass, Strength and Sarcopenia Across Severity

Quadriceps muscle thickness, STAR and handgrip strength did not differ significantly across OSA severity categories (all Kruskal–Wallis *p* > 0.05; Table 2). CST time showed a statistically significant gradient across severity strata (*p* = 0.047), though no individual pairwise comparison reached significance after Bonferroni correction. Sarcopenia prevalence was 21.4%, 30.8%, and 34.9% in mild, moderate and severe OSA respectively, with no statistically significant trend (Cochran–Armitage *p* = 0.237). Low STAR was present across all strata (89.3%, 79.5% and 83.7%, respectively), with no significant trend (Cochran–Armitage *p* = 0.615). Overall, 92 of 110 patients (83.6%) had low STAR, 30 (27.3%) had low handgrip strength, 22 (20.0%) had prolonged CST, and 33 (30.0%) met ISarcoPRM criteria for sarcopenia.

**TABLE 2:** Sarcopenia‐related parameters by OSA severity (*n* = 110).

Parameter	Mild (*n* = 28)	Moderate (*n* = 39)	Severe (*n* = 43)	p*	Pairwise (Bonferroni‐adj)
Quadriceps MT, mm	30.86 ± 6.89	32.70 ± 8.81	30.73 ± 5.97	0.749	n.s.
STAR	0.91 ± 0.25	0.98 ± 0.32	0.90 ± 0.24	0.495	n.s.
Handgrip strength, kg	31.68 ± 9.53	31.10 ± 11.09	29.81 ± 8.77	0.731	n.s.
CST time, sec+	7.76 ± 2.90 [6.75]	8.80 ± 3.73 [7.38]	9.82 ± 4.35 [8.13]	0.047	n.s.
Low STAR	25 (89.3)	31 (79.5)	36 (83.7)	CA: 0.615	—
Low handgrip strength	6 (21.4)	11 (28.2)	13 (30.2)	CA: 0.432	—
Prolonged CST ≥ 12 s	3 (10.7)	9 (23.1)	10 (23.3)	CA: 0.228	—
Sarcopenia	6 (21.4)	12 (30.8)	15 (34.9)	CA: 0.237	—

*Note:* Data are mean ± SD [median] or *n* (%). *Kruskal–Wallis for continuous variables; Cochran–Armitage (CA) trend test for binary outcomes. +CST reported with median given the right‐skewed distribution.

Abbreviations: CST, chair stand test; MT, muscle thickness; n.s, not significant; STAR, Sonographic Thigh Adjustment Ratio.

### 3.3 Correlations With AHI, ODI, Age and BMI

AHI and ODI both correlated modestly but significantly with CST time (*r* = 0.209, *p* = 0.029 and *r* = 0.203, *p* = 0.034, respectively; Table 3) but did not correlate significantly with quadriceps muscle thickness, STAR or handgrip strength (all *p* > 0.05). Age correlated with all four parameters (|*r*| = 0.28–0.53, all *p* < 0.01). BMI showed a strong negative correlation with STAR (*r* = −0.671, *p* < 0.001), reflecting the mathematical relationship between body size and the STAR denominator.

**TABLE 3:** Correlations between OSA severity indices, age, BMI and muscle parameters (*n* = 110).

Variable	Quadriceps MT	STAR	Handgrip strength	CST
Age	−0.525 (< 0.001)	−0.281 (0.003)	−0.371 (< 0.001)	+0.471 (< 0.001)
BMI	+0.019 (0.847)	−0.671 (< 0.001)	−0.097 (0.313)	+0.168 (0.079)
AHI	0.023 (0.814)	−0.050 (0.606)	−0.047 (0.623)	0.209 (0.029)
ODI	0.065 (0.497)	−0.107 (0.266)	−0.063 (0.513)	0.203 (0.034)

*Note:* Significant variables are shown as bold.

Abbreviations: AHI, apnea‐hypopnea index; CST, chair stand test; MT, muscle thickness; ODI, oxygen desaturation index; STAR, Sonographic Thigh Adjustment Ratio.

### 3.4 Independent Predictors of Sarcopenia and Low STAR

In multivariable logistic regression, OSA severity was not independently associated with sarcopenia (adjusted OR 1.19, 95% CI 0.65–2.18, *p* = 0.577) or with low STAR (adjusted OR 0.58, 95% CI 0.24–1.38, *p* = 0.219; Table 4). Age was independently associated with both sarcopenia (OR 1.12 per year, 95% CI 1.05–1.19, *p* < 0.001) and low STAR (OR 1.12, 95% CI 1.01–1.24, *p* = 0.029). Higher BMI was independently associated with both sarcopenia (OR 1.10 per kg/m2, 95% CI 1.02–1.18, *p* = 0.009) and low STAR (OR 1.33, 95% CI 1.14–1.55, *p* < 0.001). Male sex was associated with higher odds of sarcopenia (OR 3.40, 95% CI 1.20–9.67, *p* = 0.022) and low STAR (OR 11.20, 95% CI 2.57–48.86, *p* = 0.001), reflecting the sex‐specific STAR cut‐offs applied. The sex‐by‐severity interaction term was nonsignificant for both sarcopenia (*p* = 0.843) and low STAR (*p* = 0.138). When AHI was modelled as a continuous variable (per 10 events/h) in place of ordinal severity, it was not independently associated with sarcopenia (adjusted OR 1.03, 95% CI 0.81–1.30, *p* = 0.827) or low STAR (adjusted OR 0.79, 95% CI 0.56–1.10, *p* = 0.163); the direction and statistical significance were concordant with the ordinal coding.

**TABLE 4:** Binary logistic regression for sarcopenia and low STAR (*n* = 110).

Predictor	Sarcopenia Unadj OR (95%CI)	*p*	Sarcopenia Adj OR (95%CI)	*p*
OSA severity (per stratum)	1.37 (0.81–2.33)	0.239	1.19 (0.65–2.18)	0.577
Age (per year)	—	—	1.12 (1.05–1.19)	< 0.001
Male sex	—	—	3.40 (1.20–9.67)	0.022
BMI (per kg/m2)	—	—	1.10 (1.02–1.18)	0.009
Diabetes mellitus	—	—	1.72 (0.65–4.54)	0.274
Hypertension	—	—	1.14 (0.39–3.33)	0.808

Predictor	Low STAR Unadj OR (95%CI)	*p*	Low STAR Adj OR (95%CI)	*p*
OSA severity (per stratum)	0.85 (0.44–1.62)	0.615	0.58 (0.24–1.38)	0.219
Age (per year)	—	—	1.12 (1.01–1.24)	0.029
Male sex	—	—	11.20 (2.57–48.86)	0.001
BMI (per kg/m2)	—	—	1.33 (1.14–1.55)	< 0.001
Diabetes mellitus	—	—	2.00 (0.46–8.75)	0.355
Hypertension	—	—	0.89 (0.24–3.28)	0.860

*Note:* Significant variables are shown as bold. Adjusted models include all listed predictors simultaneously. OSA severity coded 0 = mild, 1 = moderate and 2 = severe.

Abbreviations: CI, confidence interval; OR, odds ratio; STAR, Sonographic Thigh Adjustment Ratio.

### 3.5 Sex‐Stratified Analysis

Sex‐stratified results are presented in Table 5. No significant severity trend was observed in either sex for sarcopenia or low STAR. Among females, handgrip strength differed across severity groups (*p* = 0.020), with the lowest values in the moderate OSA group (21.29+/−4.06 kg). No such pattern was seen in males (*p* = 0.335). The sex‐by‐severity interaction did not reach statistical significance, and this finding should be regarded as exploratory given the small subgroup sizes.

**TABLE 5:** Sex‐stratified sarcopenia parameters by OSA severity.

Parameter	Mild	Moderate	Severe	*p* (KW or CA)
**Males (*n* = 60): Mild *n* = 14, Moderate *n* = 22, Severe *n* = 24**				
Quadriceps MT, mm	33.54+/−6.21	36.54+/−8.79	32.78+/−5.13	0.382
STAR	1.05+/−0.20	1.08+/−0.28	0.95+/−0.21	0.126
Handgrip strength, kg	37.36+/−9.89	38.68+/−8.49	34.75+/−8.04	0.335
CST time, sec	7.68+/−3.15	7.21+/−2.41	9.86+/−4.83	0.054
Sarcopenia	5 (35.7)	5 (22.7)	11 (45.8)	CA: 0.385
Low STAR	13 (92.9)	19 (86.4)	23 (95.8)	CA: 0.617
**Females (*n* = 50): Mild *n* = 14, Moderate *n* = 17, Severe *n* = 19**				
Quadriceps MT, mm	28.18+/−6.68	27.74+/−6.05	28.13+/−6.06	0.988
STAR	0.77+/−0.23	0.84+/−0.32	0.84+/−0.25	0.826
Handgrip strength, kg	26.00+/−4.64	21.29+/−4.06	23.58+/−4.83	0.020
CST time, sec	7.84+/−2.75	10.85+/−4.17	9.78+/−3.80	0.086
Sarcopenia	1 (7.1)	7 (41.2)	4 (21.1)	CA: 0.460
Low STAR	12 (85.7)	12 (70.6)	13 (68.4)	CA: 0.280

*Note:* Data are mean ± SD [median] or *n* (%). Significant variables are shown as bold.

Abbreviations: CA, Cochran–Armitage trend test; CST, chair stand test; KW, Kruskal–Wallis; MT, muscle thickness; STAR, Sonographic Thigh Adjustment Ratio.

* Statistically significant; interpret with caution given subgroup size.

### 3.6 Sensitivity Analysis: Exclusion of Severe Obesity

Excluding 23 participants with BMI 40 kg/m2 or above (*n* = 87 retained: mild 23, moderate 30 and severe 34) did not change any result materially. Sarcopenia prevalence was 26.1%, 23.3% and 26.5% across severity groups (*p* = 0.946). OSA severity remained nonsignificant in adjusted logistic regression for sarcopenia (OR 0.84, 95%CI 0.41–1.70, *p* = 0.627) and low STAR (OR 0.59, 95%CI 0.25–1.41, *p* = 0.234).

## 4 Discussion

This study shows that OSA severity is not independently associated with sarcopenia or muscle mass deficit after adjustment for age, BMI and sex. Instead, age and BMI are the dominant predictors of sarcopenia in this cohort. A modest but significant association between OSA severity indices (AHI and ODI) and CST time points to physical function as a more sensitive early indicator of OSA‐related decline than muscle mass per se. The near‐universal prevalence of low STAR (83.6%) reflects important limitations of population‐specific cut‐offs applied across different ethnic and anthropometric contexts.

The null finding for OSA severity and sarcopenia is consistent with existing literature that identifies ageing and obesity as the primary drivers of sarcopenia in sleep clinic populations [13, 14]. The EPISONO cohort [13] and NHANES analysis [14] used DXA‐based whole‐body measurements and found associations between sleep‐disordered breathing and adverse body composition, but neither separated the contribution of OSA severity from that of age and obesity in adjusted models. Piovezan et al. [15] in the HypnoLaus cohort (*n* = 1902) found that severe OSA was associated with low grip strength, a functional rather than muscle mass finding. Our data extend this observation: Both AHI and ODI correlated modestly but significantly with CST time (*r* = 0.209, *p* = 0.029 and *r* = 0.203, *p* = 0.034), whereas no significant correlation with muscle mass parameters was found. Sun et al. [16] showed by Mendelian randomisation that low grip strength may be causally upstream of OSA risk, which means functional associations may partly reflect pre‐existing physical deconditioning rather than OSA‐driven muscle deterioration.

The CST gradient across severity strata (mild 7.8 s, moderate 8.8 s and severe 9.8 s; KW *p* = 0.047), combined with the AHI‐CST correlation, supports the hypothesis that intermittent hypoxia and sleep fragmentation preferentially affect lower extremity functional capacity before producing measurable changes in quadriceps muscle mass. This aligns with the known sensitivity of the CST to early functional decline in ageing populations and suggests that the CST may be a more clinically relevant sarcopenia screening tool in OSA patients than STAR alone. However, no individual pairwise comparison reached significance after Bonferroni correction, and the finding should be regarded as exploratory. Regarding the broader question of early sarcopenia detection, quadriceps ultrasound and STAR may capture regional muscle mass loss before functional deficits become clinically apparent, as muscle atrophy can precede reductions in strength and physical performance in general sarcopenia progression [4, 5]. In this cohort, low STAR was present in 83.6% of participants, whereas composite sarcopenia (requiring both low STAR and functional impairment) was present in 30.0%, a pattern consistent with muscle mass deficit preceding functional decline. The near‐universal low STAR rate, however, also reflects the BMI denominator floor effect in this high‐obesity cohort, making it difficult to fully separate true early sarcopenia signal from measurement artefact in this specific population.

The reduced STAR values seen across all severity groups, without corresponding differences in muscle mass, are explained by the mechanics of the metric. STAR divides quadriceps muscle thickness by BMI; in a cohort where mean BMI is 35.1 kg/m2 (nearly 10 units above the Turkish derivation cohort [21]), the denominator alone is sufficient to push STAR below the male cut‐off of 1.4 in the large majority of participants. The strong negative correlation between BMI and STAR (*r* = −0.671, *p* < 0.001) in our data supports this interpretation. Cut‐off values derived in one population should not be applied uncritically to another with a substantially different BMI distribution. Arabian Gulf populations, where obesity prevalence frequently exceeds 70%, need locally derived STAR normative data.

The sex‐stratified analysis found lower grip strength among females with moderate OSA compared with mild and severe categories (*p* = 0.020). No corresponding trend was seen in males. The nonmonotonic pattern and the absence of a significant sex‐by‐severity interaction (*p* = 0.138) argue against a coherent severity‐dependent effect and more likely reflect the small subgroup sizes (14–19 per cell in females). The testosterone‐mediated mechanism proposed for sex‐specific sarcopenia in OSA [23] could not be tested here as hormonal data were not collected; this is a limitation and a direction for future work.

Several limitations apply. First, the cross‐sectional design does not allow causal inference about whether OSA promotes sarcopenia or whether shared factors such as ageing, obesity and physical inactivity drive both. Second, the STAR cut‐offs were derived in a Turkish community cohort [21] and have not been validated in Kuwaiti or other Arabian Gulf populations. Third, Level 3 portable monitoring systematically underestimates AHI relative to full polysomnography [24], and severity misclassification near category boundaries is possible. Fourth, hormonal data, CPAP compliance and minimum overnight oxygen saturation were not available. Fifth, sonographers were not blinded to OSA severity classification, though this limitation is inherent to single‐centre within‐cohort designs where all patients carry the same exposure label. Sixth, patients with stable asthma, interstitial lung disease or nonexacerbating COPD were not excluded unless functionally impaired; residual confounding from these conditions, which may be independently associated with both OSA and sarcopenia, cannot be excluded. Seventh, the study was designed as an exploratory within‐cohort analysis; although the 13‐percentage‐point sarcopenia difference between mild and severe OSA approached the originally planned 15% target, formal confirmatory inference was not preregistered, and all results should be interpreted accordingly. Eighth, formal intra‐ and interrater reliability of the ultrasound measurements (intra‐class correlation coefficient or standard error of measurement) was not quantified within the study cohort; a feasibility pilot was used to standardise image acquisition but does not substitute for a formal reliability analysis.

Despite these limitations, the study offers observations of value. It is among the first to apply the ISarcoPRM algorithm prospectively in a sleep clinic setting, showing that bedside muscle assessment is feasible in this population. The CST‐AHI association is a meaningful, hypothesis‐generating finding that points to functional assessment as a more sensitive early indicator of OSA‐related decline than muscle mass. The finding that age and BMI, rather than OSA severity, predict sarcopenia has practical implications: Sarcopenia screening should be directed toward older and more obese patients with OSA regardless of AHI. The near‐universal low STAR in this high‐obesity cohort cautions against overdiagnosing ISarcoPRM‐defined sarcopenia in Arabian Gulf populations until local normative data are available.

In summary, OSA severity is not independently associated with sarcopenia in this Kuwaiti cohort after adjustment for age and BMI. Physical function, measured by CST, shows a modest but significant gradient with OSA severity that muscle mass parameters do not. The ISarcoPRM algorithm is feasible for bedside sarcopenia assessment in sleep clinic populations, but STAR cut‐offs require validation in Arabian Gulf populations with high‐obesity prevalence. Future longitudinal studies incorporating polysomnography‐confirmed severity, population‐specific STAR normative data and hormonal measurements are needed to determine whether OSA contributes independently to sarcopenia risk beyond ageing and obesity.

## Author Contributions

**A.J.A.:** conceptualization, data collection, statistical analysis, manuscript writing. **R.A.:** data collection, ultrasound measurements. **M.A. and S.K.:** patient recruitment, sleep study interpretation. **S.A.:** data collection, database management. **A.A.:** data entry, literature review. **D.S.:** study supervision, manuscript review. **M.K. and L.O.:** methodology, ISarcoPRM expertise, critical revision. All authors read and approved the final manuscript.

## Funding

The authors have nothing to report.

## Ethics Statement

This study was approved by the Kuwait Ministry of Health Ethics Committee (Reference No. 2827/2025). Written informed consent was obtained from all participants.

## Conflicts of Interest

The authors declare no conflicts of interest.

## Data Availability

The deidentified dataset supporting the conclusions of this study will be made available upon reasonable request to the corresponding author; deposition in a public repository (OSF or Zenodo) is planned prior to publication.
